# Totally opportunistic routing algorithm (TORA) for underwater wireless sensor network

**DOI:** 10.1371/journal.pone.0197087

**Published:** 2018-06-06

**Authors:** Ziaur Rahman, Fazirulhisyam Hashim, Mohd Fadlee A. Rasid, Mohamed Othman

**Affiliations:** 1 Faculty of Engineering, Universiti Putra Malaysia, Serdang Selangor Darul Ehsan, Malaysia; 2 Faculty of Computer Science and Info Tech., Universiti Putra Malaysia, Serdang Selangor Darul Ehsan, Malaysia; 3 Research Centre of Excellence for Wireless and Photonic Networks (WiPNET), Universiti Putra Malaysia, Serdang Selangor Darul Ehsan, Malaysia; Southwest University, CHINA

## Abstract

Underwater Wireless Sensor Network (UWSN) has emerged as promising networking techniques to monitor and explore oceans. Research on acoustic communication has been conducted for decades, but had focused mostly on issues related to physical layer such as high latency, low bandwidth, and high bit error. However, data gathering process is still severely limited in UWSN due to channel impairment. One way to improve data collection in UWSN is the design of routing protocol. Opportunistic Routing (OR) is an emerging technique that has the ability to improve the performance of wireless network, notably acoustic network. In this paper, we propose an anycast, geographical and totally opportunistic routing algorithm for UWSN, called TORA. Our proposed scheme is designed to avoid horizontal transmission, reduce end to end delay, overcome the problem of void nodes and maximize throughput and energy efficiency. We use TOA (Time of Arrival) and range based equation to localize nodes recursively within a network. Once nodes are localized, their location coordinates and residual energy are used as a matrix to select the best available forwarder. All data packets may or may not be acknowledged based on the status of sender and receiver. Thus, the number of acknowledgments for a particular data packet may vary from zero to 2-hop. Extensive simulations were performed to evaluate the performance of the proposed scheme for high network traffic load under very sparse and very dense network scenarios. Simulation results show that TORA significantly improves the network performance when compared to some relevant existing routing protocols, such as VBF, HHVBF, VAPR, and H2DAB, for energy consumption, packet delivery ratio, average end-to-end delay, average hop-count and propagation deviation factor. TORA reduces energy consumption by an average of 35% of VBF, 40% of HH-VBF, 15% of VAPR, and 29% of H2DAB, whereas the packet delivery ratio has been improved by an average of 43% of VBF, 26% of HH-VBF, 15% of VAPR, and 25% of H2DAB. Moreover, the average end-to-end delay has been reduced by 70% of VBF, 69% of HH-VBF, 46% of VAPR, and 73% of H2DAB. Furthermore, average hope-count has been improved by 57%, 53%, 16% and 31% as compared to VBF, HHVBF, VAPR, and H2DAB, respectively. Also, propagation delay has been reduced by 34%, 30%, 15% and 23% as compared to VBF, HHVBF, VAPR, and H2DAB, respectively.

## Introduction

**Fig 1:**
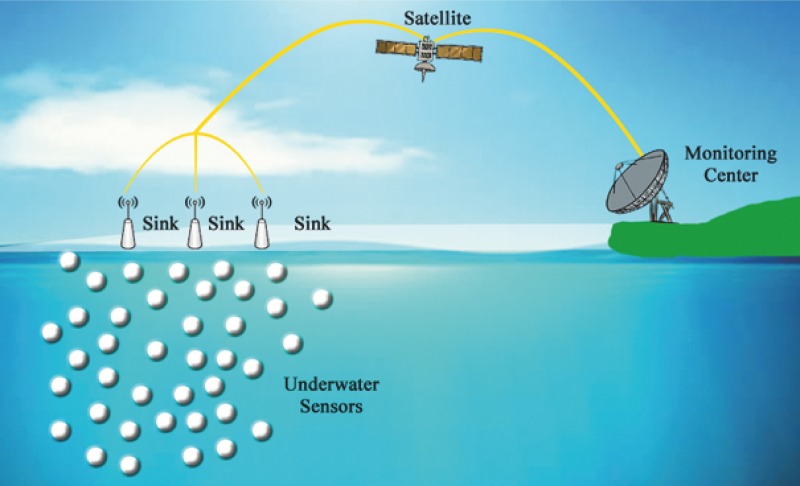
A typical UWSN environment.

In the current era, UWSN received lot of attention from the research community due to its numerous applications [1]. Applications of UWSN include underwater mineral extraction, ocean monitoring, marine and wildlife studies, geological processes on the seafloor, oil fields and navigation assistance, etc. UWSN is mainly composed of sensor nodes which are deployed inside water, and those nodes sense the environment, collect data and transfer it to one of the surface sinks. From there the data is forwarded to an onshore data center for further processing, as illustrated in Fig 1. However, due to the complex underwater environment, how to efficiently and timely send the collected data to surface sink is a challenging task [2].

Although there are some similarities between UWSN and terrestrial wireless sensor network (WSN), however UWSN exhibits some unique characteristics such as dynamic structure, narrow bandwidth, high transmission latency, high bit error rate, three dimensional network topology, limited energy and data storage are some of the particularities that differentiate UWSN from WSN [3, 4]. Radio Frequency (RF) is a standard physical layer technology for WSN however, due to rapid attenuation of radio signals in water make it infeasible for UWSN, and because of that we have to rely on the acoustic channel for communication [5].

Acoustic channels are characterized by the low bandwidth and higher propagation delay which results in an unreliable environment compared to WSN [1, 6]. Apart from acoustic signal bandwidth limitation, underwater sensor nodes can move freely with water currents which lead into a dynamic network topology. Furthermore, the links between nodes are highly error prone due to path loss, and Doppler spread [7]. Due to these characteristics, the routing protocols designed for WSN cannot be directly implemented in UWSN [6, 8]. Therefore, several novel protocols have been developed for UWSN specifically. Although some of these are very impressive in handling an individual issue but seems to be not that useful for others, that is why we do not have any standard operational routing protocol for UWSN until time.

Opportunistic Routing (OR) is one of the promising techniques proposed for wireless and ad hoc networks [9, 10], that suites UWSN environment by utilizing the broadcast nature of propagation to send data from source to destination node. In traditional routing protocols, a node is selected as a next-hop forwarder and all data is sent to the destination via designated next-hop. Whereas, OR routing protocol selects next-hop forwarder on the fly by taking advantage of the broadcast nature of the medium [11, 12].

A set of potential next-hop forwarders is selected known as candidate set (CS). Through candidate coordination, the candidate set is prioritized based on certain matrixes and best priority node is chosen to be the next-hop forwarder, called candidate forwarder (CF). CF is supposed to be the one with the lowest cost from source to destination [10]. If CF could successfully forward a packet, the rest of the CS nodes will overhear the transmission and will discard the packet, otherwise the next priority node from CS will proceed with the transmission [13].

As discussed earlier, UWSN has unreliable communication environment due to several factors and selecting next-hop forwarder on the fly is a real solution to such an environment. A packet sent to the designated next-hop may not be received, however due to the broadcast nature of the medium, the packet might be received by another neighboring node. Thus OR forward the packet to the destination via different paths and help to achieve high delivery ratio, which is one of the main challenges in UWSN [11, 14].

The central point behind OR is the use of multiple active links between the two ends and packet progresses toward the destination which results in fewer retransmissions. Reducing unnecessary duplicate data transmission results in energy conservation, which is one of the main challenges faced by UWSN [11]. In this article, we present a comprehensive review of OR protocols designed for UWSN, which are closely related to our work and then following it by our proposed scheme of the routing algorithm for UWSN.

Our proposed scheme presents a unique localization model for UWSN and based on that an OR routing protocol has been designed. In this protocol each ordinary node operations vary with its geographic coordinates, with respect to its neighboring nodes. Extensive simulations were performed to evaluate the performance of the proposed scheme in comparison to some well known routing protocols. Simulations analysis show that our proposed routing protocol extends network lifetime by improving energy efficiency and packet delivery ratio while reducing end-to-end delay and propagation deviation factor.

The remainder of this article is organized as follows: A brief overview of some existing protocols closely related to our work has been presented in the next section, followed by our proposed scheme in the upcoming section. The proposed scheme is evaluated analytically for energy consumption in the following section, whereas the simulation setup and detailed analysis of the proposed scheme is discussed in details, before the article is concluded in the last section.

## Related work

In this section an overview of some of the existing protocols, closely related to our work has been given.In this set of protocols nodes geographic position or coordinates are used as a matrix to select candidate set and on behalf of that routing decision is made to forward data from source to sink node. We present a comprehensive review of some of these protocols with their pros and cons.

### Vector Based Forwarding (VBF)

VBF is a stateless, geographic, reliable routing protocol designed specifically for UWSN [15]. VBF assumes that position of nodes is known, and virtual pipe (vector) is created between the two ends, through which data packets are forwarded in multi-hop fashion towards the surface sink. In VBF nodes lying within the virtual pipe are the potential next-hop forwarder and only they can forward the data. Thus, fewer nodes are used for data forwarding, which reduces energy utilization in comparison to traditional routing algorithms.

In VBF, once a node receives a packet, it will check whether it lies within the forwarding vector. If it is in the routing vector, it will embed its position to the header of the packet and will forward it otherwise it will discard the packet. A pre-defined distance threshold ‘w’ is used to fix the width of routing pipe between source and destination. To control duplicate packet transmissions, a self-adaptation algorithm has been proposed in VBF. With the help of this algorithm timer based approach is used to define desirability factor, which shows a node propinquity to the previous forwarder. Thus higher the desirability factor of a node the less time it will wait to forward the packet. To make the protocol reliable against packet loss in VBF more than one candidate may forward the packet along redundant paths.

VBF claims to be scalable, reliable and stateless routing protocol, however, in a low dense or high mobile network it is possible that there may be few or no node in the virtual pipe to forward data which will degrade the performance of the network. In the worst case scenario, there may be other nodes outside the virtual pipe that can deliver the packet to the destination but they will not take part in data forwarding because they are not in close proximity to the virtual pipe. Furthermore, nodes located within virtual pipe are used more frequently for packet transmission, which leads to energy depletion of those nodes.

### Hop-By-Hop Vector Based Forwarding (HH-VBF)

Defining one fixed virtual pipe from source to destination may cause less packet to be successfully received at the sink especially in the sparse network. In [16] HH-VBF was introduced to improve the performance of VBF by setting a routing pipe between each and every hop as the packet progresses towards sink node. In another word, there is a virtual pipe from each node towards a sink.

Once a node receives a packet, and it finds itself to be the candidate forwarder, it will create its virtual pipe and forwards the packet. Thus, there is a higher chance of packet delivery to the destination through different nodes in each virtual pipe.

Although HH-VBF improves the performance of VBF significantly, however, it faces some severe limitations such as duplicated packet transmission. Apart from that both VBF and HH-VBF shares the problem of void nodes, thus, if a node has data to send but it does not find any forwarding node in a closer proximity to the virtual pipe towards the destination, then it discards the packet.

### Depth Based Routing (DBR)

Depth based, any-cast opportunistic routing protocol has been proposed for UWSN in [17]. DBR assumes that each node is equipped with an inexpensive depth sensor which measures the depth of a node. This depth information is used as a matrix to decide whether to forward a packet or not. Thus, nodes which are closer to water surface are more likely to receive and forward data. Similar to VBF and HH-VBF, DBR is also a receiver based opportunistic routing protocol, which uses depth of a node as an OR matrix.

In case of DBR, when a sensor node has data to send, it calculates its depth value and puts that value into a packet header and broadcast it. All the receiving nodes will compare their depths with the depth in the packet header. Nodes with lower depth (closer to water surface) are potential forwarder, whereas nodes with higher depth will just discard the packet. In order to control duplicate packet transmission, DBR uses timer based coordination. Thus, a candidate node with the lowest depth gets the highest priority for data forwarding and holds the packet for a minimum duration. Once the timer expires, the node will proceed with data forwarding unless it hears the same packet transmission from another neighboring node. Apart from that depth threshold is added to data packet header which results in the reduction of duplicate transmission.

DBR is claimed to be a scalable routing protocol which does not need any control message transmission to obtain nodes depth information. However, it does not have any recovery mechanism and same like VBF and HH-VBF once a packet reaches to void node the packet is discarded after few trials. Thus, it causes a local maximum problem in the sparse scenario. Apart from that, packet delivery ratio depends on the value of depth threshold, larger the depth threshold may leave fewer nodes for data forwarding, which results in lower packet delivery ratio and if the depth threshold is set to be small, many nodes may be eligible for packet forwarding, which leads to duplicate transmission.

### Void Aware Pressure Routing (VAPR)

Void node problem is one of the greatest setback for greedy based routing protocol. The communication void problem encounters if there exist no neighboring node to forward data packet towards a sink. Such a problem is very common in greedy routing protocols. To tackle this issue an OR based routing protocol named VAPR has been proposed in [18]. VAPR adopts multi-hop architecture, whereas ordinary nodes are randomly deployed and can move freely with water currents.

VAPR performs its operation within two phases i.e. enhanced beaconing and opportunistic data forwarding. Periodic beaconing messages are sent by sink nodes, which carry information like depth, hop count, and direction of the current node. These beacon signals are used to identify the direction of each node in a heuristic manner; these beacon messages are kept on updating and rebroadcasting by each receiving node. If a beacon is received from a lower depth node then the direction of the update will be upward; otherwise, it updates downward.

During the opportunistic data forwarding phase, the direction of neighboring nodes is used to forward data towards sink nodes. VAPR holds the information until two-hop adjacent nodes and tries to avoid packet to be trapped into communication void region.

Although VAPR strives to bypass the communication void region, while reducing communication overhead and end-to-end delay which turns out to be its biggest advantage. However, it suffers few many shortcomings such as packet can be forwarded in either upward or downward direction which excludes horizontal transmission that might be useful in certain scenarios. In VAPR, nodes are forced for calculating its distance to neighboring nodes and rebroadcast this information frequently. That significantly increase network overhead.

### Hop-to-Hop Dynamic Addressing Based Routing Protocol (H2DAB)

H2DAB is a localization free novel UWSN routing protocol, proposed in [19]. This protocol performs its operation within two phases i.e. setup phase and data forwarding phase. In the first phase of operation dynamic hop IDs are assigned to deployed nodes while in second and last phase data are forwarded towards sonobuoys by using these hop IDs as an OR matrix.

H2DAB divides the area under observation into several layers, vertically from water surface into the bottom of an ocean. Each and every sensing node must belong to one of these layers. Each layer receives data from a lower layer and after data aggregation forwards it toward the surface sink. An analytical model has been formulated to calculate energy consumption.

To solve the issue of continuous node movement with water current; H2DAB uses dynamic addresses for each sensor node. Thus, a sensor node will get new ID on changing its position every time. Nodes nearer to the surface will acquire a smaller ID; this keeps on extending as node move toward the bottom of an ocean.

This process precedes as follows, Hello packets generated by surface sinks are used to assign dynamic addresses to ordinary nodes. Every node receiving this packet gets a hop ID and then forwards it to nodes located at the higher depth until it reaches a certain number of defined nodes. Once all nodes are localized, the first phase comes to an end.

The second phase starts with a node having a data packet to send. It will broadcast an inquiry request and all the receiving nodes with smaller hop IDs are the potential forwarders, and they will acknowledge this message by sending an inquiry reply. Based on certain matrixes the best-suited node will be selected as a candidate forwarder and the data packet will be forwarded to it. This process continuous until the data packet is received by any of the deployed sonobuoys. Sonobuoys, deployed at water surface have both acoustic, and RF modem and they have the luxury of using RF modem to transmit data to the onshore data center with a much higher bandwidth and less propagation delay.

Although it seems like H2DAB have somehow solved the issue of finding node location. However, Hop Id may not be that useful in routing protocol as much the node complete location information. Moreover, H2DAB uses too many broadcast messages for assigning IDs to nodes and forwarding data packets.

### Directional Depth Based Routing (D-DBR)

D-DBR is single sink based routing protocol which is proposed to forwards data packets through optimal path to the sink node [20]. The only sink node is deployed on ocean surface and supposed to have a high battery power while the ordinary nodes are deployed at depth of water. D-DBR uses Time of Arrival (ToA) techniques for data forwarding mechanism whereas holding time function and angle holding time function is used for route creation and maintenance.

D-DBR faces some severe limitations including low delivery ratio in sparse area and lack of proper methodology for saving ordinary nodes energy. Moreover, D-DBR has no mechanism for avoiding void region, which is one of the main problems in underwater environment.

### Cluster Vector Based Forwarding (CVBF)

A cluster based routing protocol has been proposed in [21], called Cluster Vector Based Forwarding (CVBF). CVBF improves data delivery ratio and reduces end to end delay by considering the sparse and dense area of the network. In CVBF the whole network is divided into different regions, called clusters. In every cluster one node is selected as a virtual sink based on its geographic location. Each ordinary node within a cluster sends its data to its respective virtual sink via virtual pipe, similar to VBF. The radius of the virtual pipe is equal to the transmission range of a node. Once the data is collected by virtual sink, it performs data aggregation function on the received data and transmit it by a single hop to the sink node deployed on water surface.

Although the authors of the CVBF claimed that the approach and methodology defined in CVBF is better than VBF and HH-VBF, however it suffers few many shortcomings such as development of multiple clusters and multiple routing pipes, which is not an easy task in the harsh underwater environment. Moreover, due to continues node movements a node may move away from its routing pipes and will drop the packets, which will adversely affect the data delivery ratio.

A summary of the existing protocols is given in Table 1.

**Table 1:** Summary of the protocols.

Protocol	OR Matrix	Candidate Selection	Candidate Coordination	Receiver/ Sender Based	Pros.	Cons.
VBF	GeoGraphic	×	Timer Based	Receiver Based	Scalable, Robust	Duplicate Tx, Void Region
HH-VBF	GeoGraphic	×	Timer Based	Receiver Based	Scalable, Robust	Duplicate Tx, Void Region
DBR	Depth	×	Timer Based	Receiver Based	Simple Algorithm	Duplicate Tx, Void Region
VAPR	Depth	✓	Timer Based	Sender Based	No Void Region	Complex Algorithm
H2DAB	Depth	✓	Timer Based	Receiver Based	Scalable, Energy Efficient	Complex Algorithm, Void Region

To the best of our knowledge, there is no published work in UWSN where node operations vary with its geographic coordinates, with respect to its neighboring nodes. Apart from that, most of the published work in this field focuses either on nodes localization or routing algorithm. Our proposed scheme is the first of its nature which introduces a unique network architecture for UWSN and based on that we proposed a routing algorithm for underwater mobile sensor networks.

## Totally opportunistic routing algorithm (TORA)

In this paper, we propose a hierarchical localization scheme, and based on that we designed a novel anycast, receiver based opportunistic and geographical routing protocol known as TORA. In TORA nodes are localized with the help of trilateration and TOA, and node location information along with its residual energy is used to find the best available forwarding node in a closer proximity to the destination. Thus multiple short and active links are combined to transmit data to the sink. The proposed scheme performs its operation within three phases namely, nodes localization, candidate forwarder selection and data transmission.

### Nodes localization

In our proposed scheme two types of nodes are used i.e., surface sonobuoys and ordinary sensing nodes. Sonobuoys drift on ocean surface, whereas ordinary nodes are randomly deployed in the ocean, as can be seen in Fig 1. These sonobuoys are equipped with a common GPS, and they are supposed to get their exact location from the satellite. Ordinary nodes can either be a DTN (Double Transmission Node) or STN (Single Transmission Node). At the very first stage, ordinary nodes which are in transmission range of sonobuoys are declared to be STNs. These nodes get its location with the help of sonobuoys and assist the rest of nodes in localization, whereas DTNs are supposed to be not within transmission range of sonobuoys, however, they can communicate with STNs to estimate their position in the network.

**Fig 2:**
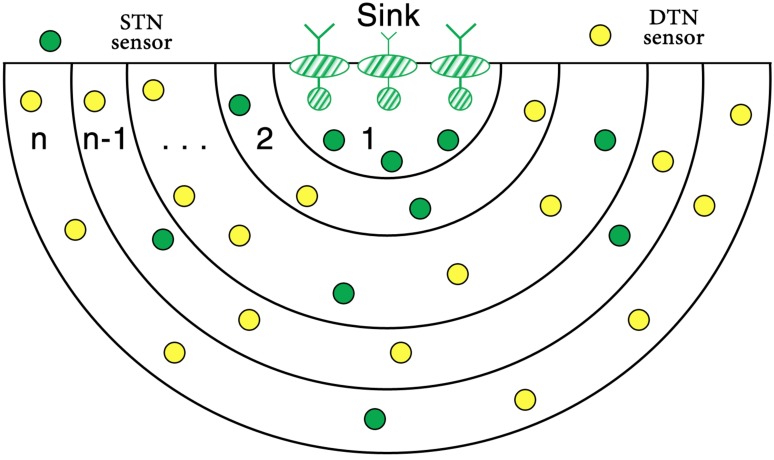
Layering structure in TORA at node localization phase.

Our proposed scheme based on multi-sink architecture and three or more sink nodes should be deployed at the water surface, as shown in Fig 2. Ordinary nodes are equipped with a pressure sensor and with the help of that they can find their depth information. Once we know the depth of a node we can project the 3-dimensional localization problem into 2-dimensional and can predict a node location coordinates by range-based localization scheme.

In our proposed scheme, we employ range based estimation followed by trilateration to get ordinary node position. For trilateration, each node must get location information from at least 3-reference nodes. The process proceeds as follows; Node localization phase starts with sink hello message. Sinks deployed at water surface periodically send hello messages. This message contains certain parameters that help ordinary nodes in localization such as {*Packet Type, Node ID, Node Status, Location Coordinates, Confidence Value, Layer ID, Distance to Sink*}.*Packet Type* specifies whether the packet is initiated by Sonobuoys or ordinary node and whether it is a data or control packet. *Node ID* is a unique ID to each node, whereas *Node Status* represents the type of node, whether it’s a sonobuoy, STN, DTN or non-localized node. *Location coordinates* are the absolute location of a node. The *Confidence Value* is a normalized form of location error, which will be explained later in details. *Distance to Sink*, *Layer ID*, and *Confidence Value* will be zero for sink hello packet; these parameters will then be utilized by STN to further the localization process.

All those nodes which directly receive Hello message from any sink node are declared to be STN. STN employs sink Hello message to calculate its distance to the sink by utilizing Time of Arrival (TOA). With the help of TOA, we can estimate the distance between two nodes by using the signal transmission time. If a packet is sent by acoustic signal at time *t1* and it reaches a sink by time *t2* then the distance between the source and sink node can be calculated as,

$$\begin{array}{c} Dist.2sink=V(t2-t1) \end{array}$$ (1)

where *V* is acoustic signal propagation speed i.e. 1500m/s.

Eq (1) is used to find distance between sender and receiver. Once a node knows its distance to three of reference nodes it uses triletaration to find out its location coordinates. In order to find a node exact location coordinates the three reference nodes selected should be such that the three planes formed by overlapping of three spheres should intersect at one point,

$$\begin{array}{c} {\left(u-x_{1}\right)}^{2}+{\left(v-y_{1}\right)}^{2}+{\left(w-z_{1}\right)}^{2}=R_{1}^{2} \end{array}$$ (2)

$$\begin{array}{c} {\left(u-x_{2}\right)}^{2}+{\left(v-y_{2}\right)}^{2}+{\left(w-z_{2}\right)}^{2}=R_{2}^{2} \end{array}$$ (3)

$$\begin{array}{c} {\left(u-x_{3}\right)}^{2}+{\left(v-y_{3}\right)}^{2}+{\left(w-z_{3}\right)}^{2}=R_{3}^{2} \end{array}$$ (4)

where (*x*_1_, *y*_1_, *z*_1_), (*x*_2_, *y*_2_, *z*_2_) and (*x*_3_, *y*_3_, *z*_3_) are coordinates of three reference nodes whereas (*u*, *v*, *w*) are coordinates of a non-localized nodes and *R*_1_, *R*_2_, *R*_3_ are distances between a non localized node to three reference nodes. In our proposed scheme we assume that depth of a sensor node can be found out with the help of pressure sensor. The relationship between pressure and depth and their respective calculation can be found in details in [22]. The distance to reference nodes are already calculated with the help of TOA and *z*–*axis* of a node can be found out with the help of pressure sensor. Thus, we have two unknown variables; *x*, *y* and three equations. We can now solve these equations to find the coordinates of non-localized STN.

Once STNs around sonobuoys are localized; then its confidence value is set to be one i.e. *ξ* = 1. The next stage in localization phase is to localize the rest of network nodes recursively. This process proceeds as follows:

Each STN periodically broadcasts sink Hello message; {*Packet Type, Node ID, Node Status, location Coordinates, Confidence Value, Layer ID, Distance to Sink*}. Each non-localized node also broadcasts localization messages, which are restricted to a certain number by a counter *n* and a threshold *N*. A counter *m* is used by each non-localized node to store the number of STNs to which it knows the distance.

**Fig 3:**
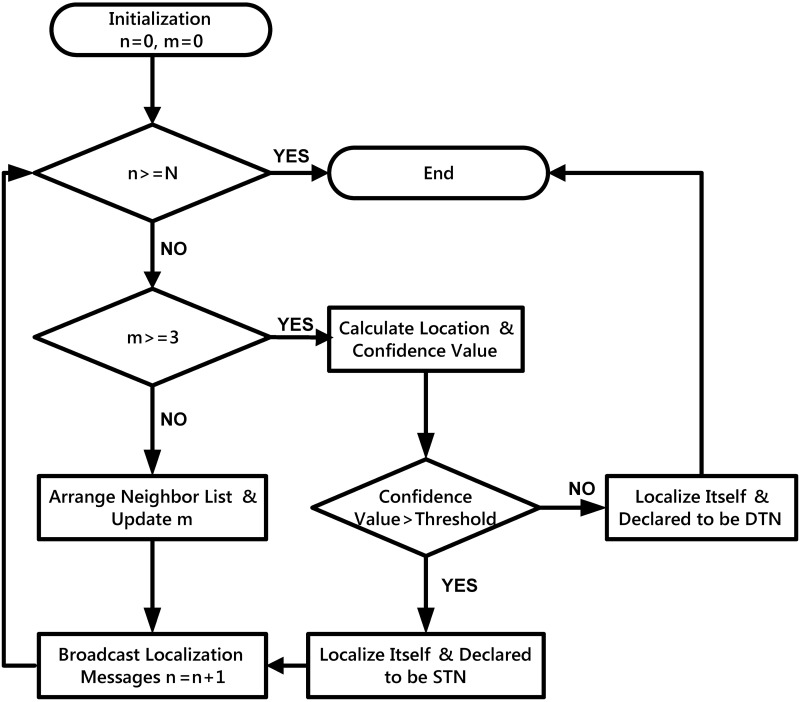
Nodes localization in TORA.

Once a non-localized node gets to know location coordinates and its distance to three STN. It utilizes TOA and triletarion to localize itself; furthermore, it can become STN if its confidence value is more than defined threshold value, otherwise it will be marked as DTN, as can be seen in Fig 3.

In our proposed scheme location error is represented by *δ* and it can be calculated by using the following formula,

$$\begin{array}{c} \delta =\sum_{k=1}^{3}(u-x_{1}{)}^{2}+{\left(v-y_{1}\right)}^{2}+{\left(w-z_{1}\right)}^{2}-l_{k}^{2} \end{array}$$ (5)

where (*u*, *v*, *w*) are the estimated coordinates of a non-localized node, (*x*, *y*, *z*) are the coordinates of reference nodes and *l*_*k*_ is the distance between non-localized node and reference nodes, which is calculated with the help of Eq (1). In order to define node’s status we utilize the normalized form of location error, called confidence value (*ξ*). Confidence value can be calculated by the following equation.

$$\begin{array}{c} \xi =\{\begin{array}{cc} 1 & \text{First}\;\text{Layer}.\\ 1-\frac{\delta }{\sum_{k=1}^{3}{\left(x-x_{k}\right)}^{2}+{\left(y-y_{k}\right)}^{2}+{\left(z-z_{k}\right)}^{2}} & \text{Otherwise}. \end{array} \end{array}$$ (6)

We have defined a threshold value, *φ*. If for an unknown node *ξ* is greater than *φ*, then its status is set to be STN and it can become a reference node; otherwise, it will become a DTN. Once all nodes are localized, and they are differentiated by their status of being STN or DTN, the node localization phase comes to an end.

### Candidate forwarder selection

From in-depth literature review we concluded that receiver based opportunistic routing protocols suit well the UWSN environment [23]. Selecting the best available forwarding node is the most important and challenging task for any routing protocol. In our proposed scheme the next forwarder is selected on the fly based on certain matrices.

Once a sender broadcast a data packet, all the receiving nodes are regarded as potential candidate forwarders. To select the best forwarding node we prioritize these nodes and allow the best priority node to proceed with packet transmission by assigning a small holding time to it. All the remaining nodes who received this same packet will wait for a certain amount of time until they overhear the same packet transmission from another node or their holding timer for the packet expires. If a node overhears transmission of a packet with the same sequence number before its timer expires, then it will drop the packet; otherwise, it will proceed with the transmission as soon as its timer expires.

In our proposed scheme a node residual energy and its distance to sink is used as a candidate matrix. Thus, a node with higher residual energy and closer to sink is highly likely to be the next forwarder. Because such a node carries smaller holding time, as can be seen,

$$\begin{array}{c} H_{Time}=(1-\frac{E_{r}}{E})*[1-(\frac{D_{S,F}-D_{S,S}}{R})]*T_{Delay} \end{array}$$ (7)

where *H*_*Time*_ is the holding time for any receiving node and *E* and *E*_*r*_ is a node initial and residual energy respectively. *D*_(*S*,*F*)_ is the distance between a source and forwarding node, whereas *D*_(*S*,*S*)_ is the distance between source and the sink node, *R* is the maximum transmission range, and *T*_*Delay*_ is a predefined maximum delay. *T*_*Delay*_ is set to be in such a way that all nodes in forwarding set could hear the transmission from highest priority node before they proceed with their transmission of the same packet.

Once a node receives a data packet, it will compare its depth with the one mentioned in the packet header. If the node is locating at a higher depth, then it will discard the packet, whereas nodes locating at lower depth will proceed with calculating its distance to the source using TOA and will calculate its holding time for that particular packet. A node with higher residual energy and closer to sink will get higher priority to relay the packet, as shown in the simple pseudo codes of Algorithm 1.

**Algorithm 1** Source is ready to send data packet

Receiving Nodes: CS has received the data packet

**if** Receiving Node Depth > Source Node Depth **then**

 Drop the data Packet

**else**

 Calculate Holding Time for the packet

**end if**

**while** Holding Timer Expires **do**

 Overhear the channel

 **if** Packet with the same sequence number overheard **then**

  Drop the packet

 **else**

  Forward the packet

 **end if**

**end while**

### Data transmission

TORA is designed to avoid horizontal transmission, reduce end to end delay, overcome the problem of void node and maximize throughput and energy efficiency. Data transmission phase plays a vital role in achieving these objectives. This phase starts once a node has data packet ready to send. This packet should be transmitted to one of the sonobuoys in a multi-hop fashion. Each data packet consists of certain parameters such as {*Packet Type, Sequence Number, Node Id, Node Status, Location Coordinates, Layer ID, Distance to Sink, Hop Count, Data Payload*}; which helps in selecting a candidate forwarder. In our proposed scheme a candidate forwarder is decided on the fly, and it is supposed to lead the packet toward the destination on the best available routing path.

In our proposed scheme the number of acknowledgment for a data packet varies from 2-hop *Ack* to zero *Ack*. The number of *Ack* based on the status of sender and receiver node. The 2-hop *Ack* is used to improve data delivery ratio and handle void node issue, whereas zero *Ack* is used to reduce end to end delay and number of control messages transmission.

Communication void region is serious issue to UWSN and it degrades the performance of the network maximally. Communication void region occurs whenever a node with data does not have any neighboring node to forward the data towards destination. Whenever a data packet stuck with such a void node, an alternate routing path should be determined or it will be discarded. Data packets discarded at void nodes will affect the network performance adversely, which is already impaired by packet loss due to noisy and unreliable nature of the underwater wireless communication.

**Fig 4:**
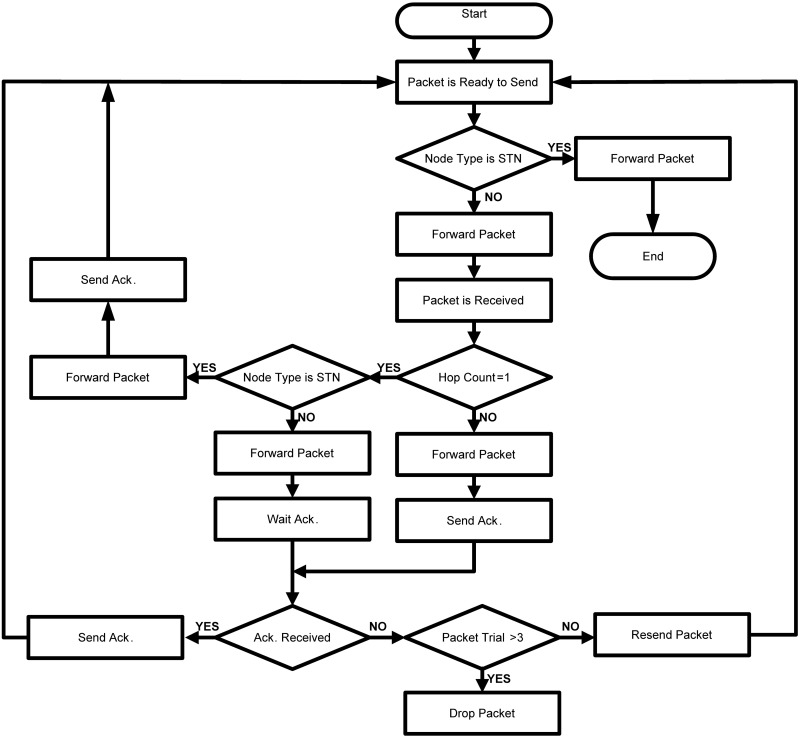
Data transmission phase in TORA.

In our proposed scheme we utilize control messages such as *Ack*, which are smaller in size and exchanged regularly to avoid the void nodes and if a packet stuck within communication void region then it helps to determine an alternate path to the destination. The 2-hop *Ack* is used to make sure that the packet has been traveled for 2 hops and if the *Ack* is not received then the packet is supposed to be stuck with a void node and a new path is determined. The new path may lead the packet with more hops and may cost energy but it guarantees packet delivery and reduces packet lost, as shown in Fig 4.

TORA divides ordinary sensing nodes into two subcategories i.e. STN and DTN. STNs are nodes which can make direct contact with sonobuoys, or their confidence value is higher than defined threshold value. Thus, each STN is supposed to have at least three nodes in its transmission range from the upper layer with the least possible localization error.

If the sender is STN, it will send the data packet without been worrying about its reception at the other end. The first layer of the network is composed of STNs, which aggregate all network data and forwards it to surface sinks. In order to reduce control message transmission these nodes do not demand any *Ack* As these nodes are either in direct contact with sonobuoys or have at least three reference nodes in the upper layer within transmission range. Thus, there are maximum chances of any packet forwarded by these nodes to be received by nodes residing at the upper layer.

TORA is an opportunistic routing protocol which utilizes broadcast nature of the medium. In most of the cases, there will be more than one potential forwarder. The list is prioritized based on certain matrices, called candidate set. In the prioritized candidate list if the most suited node fails to forward a packet because it moves into a void region and could not find any neighboring node to forward the packet then a node with next in priority list will proceed with packet transmission. Apart from that node status is refreshed after a certain period; thus, there are rare chances of nodes to become void.

On the other hand, if the sender is DTN, then a packet transmission may be backed by a single hop or 2-hop *Ack*, the type of *Ack* depends on the status of receiving node. If receiving node is STN, it will send an *Ack* soon after forwarding the packet however, if the receiving node is DTN as well, then it will hold the *Ack* for a defined period and will proceed with transmitting the packet by adding one to the hop counter. Any node receives the packet in which hop-counter parameter is 2, and this node starts to forwarding it; it is must to send back *Ack*. The *Ack* is sent back 2-hops the same path in the reverse direction and the hop counter is reinitialized.

A timer is set on the forwarding node soon after the packet leaves the node for transmission. If the forwarding node does not receive *Ack* for the packet until the timer expires. The packet is supposed to be lost, which may be during transmission or the forwarding node is having no neighbor in the upper layers to receive the packet. In this case the forwarding node will drop the packet and will not send *Ack* back to the source.

On expiration of source timer, it will start retransmission of the same packet. If the same forwarder receives the packet again, the holding time for the forwarder will be set to double of the last time. Thus, other nodes in the neighboring will have lower holding time and will proceed with packet transmission. So we use packet delivery probability as criteria for selecting next hop forwarder, which helps us avoid a single forwarder to make multiple attempts for transmitting the same packet.

Once a source receives 2-hop *Ack* for a packet, the packet is considered to be successfully transmitted. Otherwise, it will do a couple of more tries for packet transmissions before it permanently drops the packet, as can be seen in Fig 4.

Thus, 2-hop *Ack* is used to ensure packet transmission and handle the local maximum problem.

$$\begin{array}{c} HT-F=2*[(\frac{R}{V})+T_{Delay}] \end{array}$$ (8)

where *HT* − *F* represents holding time for a forwarding node to wait until receives *Ack* from the 2nd Hop node, *R* is the maximum transmission range for a node and *V* is acoustic signal propagation speed i.e. 1500*m*/*s* and *T*_*Delay*_ is a system parameter that represents a predefined maximum delay. *T*_*Delay*_ is set to be in such a way that all nodes in forwarding set could hear the transmission from highest priority node before they proceed with their transmission of the same packet. On the other hand holding time for a source node is set to be double of the *HT* − *F* to receive 2-hop *Ack* or to declare the packet is lost and proceed with re-transmission.

$$\begin{array}{c} HT-S=2*HT-F \end{array}$$ (9)

where *HT* − *S* shows holding time of a sender, which is set to be double of forwarder’s holding time. Once this timer expires and sender receives no *Ack*, the packet is assumed to be lost and the sender will start re-transmitting the same packet.

## Analytical model for energy consumption

In our proposed scheme *N* number of nodes are randomly deployed within a monitoring area *A*. At the end of the localization phase, these nodes are divided into different layers. Each node has an initial energy *ϵ* which is utilized by sending and receiving data and control packets. The only control packet used in data transmission phase is the *Ack*, which consumes very little energy as compared to the data packet.

We assume that nodes have already been localized, as that process is done once for a long time. In our proposed scheme *E*_*TX*_ and *E*_*RX*_ are transmission and reception energy respectively. The monitoring area is divided into *m* layers, and each layer consists of *n* nodes. If the whole network produces *D* packets, then each node is supposed to generate *D*/*N* packets, which is represented by *K*. Each layer receives data produced by a node residing at a lower layer, combine that with its data and forwards it towards sink node. In this section we evaluate the proposed scheme for both best and worst cases, regarding energy consumption.

### Worst case scenario

The proposed scheme is supposed to consume more energy if data is generated at bottom layer and it travels to the first layer all the way through DTNs only. Data passes through DTNs generates 2-hop *Ack*, which helps to avoid local maximum problem however, sending 2-hop *Ack* for each packet results in high energy consumption. Thus there is a trade off between energy efficiency and avoiding the local maximum problem.

For worst case scenario; we suppose a packet is generated at each layer, which is forwarded by DTN at each hop until it reaches the first layer. Packets generated or collected at first layer could be directly sent to a sink node with zero *Ack*.

$$\begin{array}{c} E_{\left(1\to S\right)}=E_{TX}\\ E_{\left(2\to S\right)}=(E_{RX}+3E_{TX})+(E_{RX}+E_{TX})\\ E_{\left(3\to S\right)}=(E_{RX}+4E_{TX})+(2E_{RX}+3E_{TX})+(E_{RX}+E_{TX})\\ E_{\left(4\to S\right)}=(E_{RX}+5E_{TX})+(2E_{RX}+4E_{TX})+(2E_{RX}+3E_{TX})+(E_{RX}+E_{TX})\\ E_{\left(5\to S\right)}=(E_{RX}+6E_{TX})+(2E_{RX}+5E_{TX})+\cdots +(2E_{RX}+3E_{TX})+(E_{RX}+E_{TX})\\ \vdots \\ E_{\left(m\to S\right)}=(E_{RX}+(m+1)E_{TX})+(2E_{RX}+m.E_{TX})+(2E_{RX}+(m-1)E_{TX})+\cdots +(2E_{RX}+3E_{TX})+(E_{RX}+E_{TX}) \end{array}$$

The first layer can transmit its data directly to a sink node that costs *E*_*TX*_ per packet; however all the other layer’s nodes have to go through the first layer to reach a sink. Once a node from the 2nd layer broadcast a packet, it will be acknowledged and forwarded to a sink by the 1st layer. Thus 2nd layer consumes *E*_*TX*_ for packet transmission whereas *E*_*RX*_ for *Ack* reception. On the other hand, to forward the 2nd layer packet the 1st layer consumes *E*_*RX*_ for receiving the packet and 3*E*_*TX*_ for transmitting this packet; along with its own data packet to a sink and for sending back *Ack* to the source.

Thus the 1st layer has to process all the information received from bottom layers. If each node of a layer generates *K* data packets, then the above equation can be expressed as,

$$\begin{array}{c} \begin{array}{c} E_{\left(m.K\to S\right)}=\left(E_{RX}+\left(m+1\right)K.E_{TX}\right)+\left(2E_{RX}+m.K.E_{TX}\right)\\ +\left(2E_{RX}+\left(m-1\right)K.E_{TX}\right)+\cdots +\left(2E_{RX}+K.3E_{TX}\right)+\left(E_{RX}+K.E_{TX}\right) \end{array} \end{array}$$

The above equation shows that the 1st layer faces maximum energy consumption i.e.(*E*_*RX*_ + (*m* + 1)*K*.*E*_*TX*_). Whereas layer *m* consumes the least of energy (*E*_*RX*_ + *K*.*E*_*TX*_) for forwarding *K* data packets.

We can calculate the energy consumption of *i*^*th*^ layer when it generates its own data and forward the data it receives from the lower layer as following.

$$\begin{array}{c} E_{i}=\left(\left(m-i+1\right)K.E_{TX}\right)+\left(K.E_{TX}+2E_{RX}\right) \end{array}$$ (10)

Replacing *K*.*E*_*TX*_ by λ.

$$\begin{array}{c} E_{i}=\left(m-i+1\right)\lambda +\lambda +2E_{RX} \end{array}$$ (11)

$$\begin{array}{c} E_{i}=\left(m-i+2\right)\lambda +2E_{RX} \end{array}$$ (12)

From which we can calculate lifetime of *i*^*th*^ layer as,

$$\begin{array}{c} T_{i}=\frac{n.\epsilon }{\left(m-i+2)(\lambda \right)}+2E_{RX} \end{array}$$ (13)

If we know the lifetime of a layer and the average number of nodes within that layer, then we can calculate the lifetime of a node at that layer as,

$$\begin{array}{c} T_{\frac{i}{n}}=\frac{\epsilon }{(m-i+2)\lambda }+2E_{RX} \end{array}$$ (14)

### Best case scenario

For our proposed scheme the best scenario is that the data packet is forwarded all the way by only one STN at each layer. Thus, there is no need of acknowledgment and packets travel only in the upward direction towards sink nodes. Suppose we have *m* layer and each layer have *n* nodes, thus the total energy consumed by a candidate set *CS* with *n* nodes for forwarding one packet can be calculated as,

$$\begin{array}{c} E\left(CS,n\right)=E_{TX}+n.E_{RX} \end{array}$$ (15)

However, some of the nodes will drop packet soon after reading the header of the packet. By removing these nodes from each layer, we can calculate the amount of energy that the new forwarding set will consume as following,

$$\begin{array}{c} E\left(CS,r\right)=E_{TX}+r.E_{RX}+\left(n-r\right).E_{RE} \end{array}$$ (16)

where *E*_*RE*_ represents the amount of energy a node spent to read header of a packet. By subtracting Eq (16) from Eq (15) we get the amount of energy saved by removing (*n* − *r*) nodes from the candidate set.

$$\begin{array}{c} ES\left(CS,\left(n-r\right)\right)=\left(n-r\right).\left(E_{RX}-E_{RE}\right) \end{array}$$ (17)

Suppose *K* number of packets are generated and they have to travel *M* hops to reach a sink node. As there is no retransmission mechanism defined for STN, then by removing *N*_*AVG*_ from each hop the total energy saved can be calculated as,

$$\begin{array}{c} ES_{TOTAL}=K.M.N_{AVG}\left(E_{RX}-E_{RE}\right) \end{array}$$ (18)

If the nodes are allowed to retransmit a lost packet by implementing retransmission mechanism, then the amount of energy saved will depend on how many nodes are capable of receiving the packet and how many retransmission will be performed until the packet reaches the destination. To maximize energy saving, we would have to reduce the number of receiving nodes as well as retransmissions. However, if we decrease the number of receiving nodes, it will increase the probability of retransmissions because of frequent packet failure. A transmission is considered to be successful if at least one node from an upper layer receives the data packet without any error.

In UWSN, acknowledgment mechanism is costly in the sense of energy consumption and apart from that a node can listen to neighboring transmission, and that is supposed to be enough to suppress or drop a packet, which has already been successfully transmitted by a neighbor. That is the reason our proposed scheme introduces STNs, which are zero-acknowledgment nodes.

## Simulation setup

In this section, we evaluate the performance of TORA with a stateless geographical opportunistic routing protocol, VBF and three other closely related UWSN routing protocols, HH-VBF, H2DAB, and VAPR. All the protocols have been implemented in NS2 based simulator, called *AquaSim*1.0 [24], which is highly reliable and flexible simulator developed on NS2 to simulate the impairments of acoustic channel in UWSN.

It’s a matter of fact that the performance of routing protocol is directly affected by underlying MAC protocol. Similar to most of the previous works done in this field [17, 18], we use CSMA protocol at the MAC layer. Due to broadcast nature of the medium, neighboring nodes can over-hear the packet transmission, and they will suppress the redundant transmission if other candidates have already relayed the packet.

In our simulation, the number of nodes ranges from 100 to 500, and the number of sonobuoys is 40. The sonobuoys float on water surface and the ordinary nodes are randomly deployed within a region size of 1500*m* × 1500*m* × 1500*m*. The ordinary node can move horizontally with water currents with a speed of 2 *m*/*s* by following random walk 2*D* mobility model, which had been implemented in VBF and HH-VBF.

The maximum transmission range for all node is set to be 300 *meters*, whereas data is generated independently on each node according to Poisson process with a rate of λ packets/second, where λ ∈ {0.01,0.05}. The propagation speed for acoustic signal inside water is configured to be 1500 *m*/*s*, with a data rate of 50 *kbps*. The size of a packet may vary with the number of forwarding nodes, but its average size is 150 *B*, as can be seen in Table 2.

**Table 2:** Simulation parameters.

Parameter	Value
Simulation Software	NS2.30(Aqua-Sim-1.0)
Topography Dimension	1500*m* × 1500*m* × 1500*m*
Traffic Type	CBR
Size Of Packet	150 Byte
Transmission Frequency	25KHz
Maximum Range	300 meter (in all directions)
Simulation Time	3600 s
Initial Energy	12000 J
Number Of Nodes	{100,500}
Number Of Sink Nodes	{10,40}
Localization Message Counter	n {0,5}
Min. Number of Reference Nodes within Range	m {0,3}
Max. Localization Message Counter	N {5}
Propagation Speed of Acoustic Signal	1500 m/s
Data Generation Rate	λ Packets/Second [λ ∈ {0.01,0.05}]
Confidence Threshold	(*φ*) {0.95}
Transmission Energy Consumption	*E*_*TX*_ {2W}
Packet Reception Energy Consumption	*E*_*RX*_ {0.75W}
Packet Header Reading Energy Consumption	*E*_*RE*_ {0.25W}
Idle Listening Energy Consumption	*E*_*IL*_ {10mW}

In our proposed scheme, we set the confidence threshold (*φ*) to be 0.95 whereas *N* and *T*_*Delay*_ takes the value of 5 and 200 *ms* respectively. For VBF and HH-VBF we configure the radius of the routing pipeline to be 100 *meter*, which is one-third of the maximum transmission range, and these protocols perform the best with this range. The energy consumption for each sensor node is the combination of energy consumed during transmission (*E*_*TX*_), reception (*E*_*RX*_), packet header reading (*E*_*RE*_) and idle listening (*E*_*IL*_). The values for energy consumption were set to be *E*_*TX*_ = 2*W*, *E*_*RX*_ = 0.75*W*, *E*_*RE*_ = 0.25*W* and *E*_*IL*_ = 10*mW* for the respective sensor operation. In our simulation, the results are averaged for 20 runs, where each run lasted for 1 hour with a 95% confidence interval.

### Simulation results with different value of confidence threshold

In this section, we present simulation results of TORA for different values of confidence threshold. Confidence threshold is an important parameter of TORA which affects the energy consumption, end to end delay and packet delivery ratio of the network.

**Fig 5:**
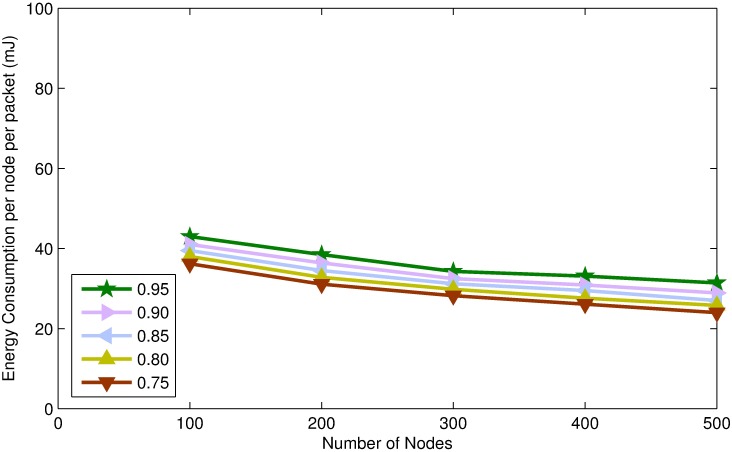
Energy consumption per node per packet vs. node density.

**Fig 6:**
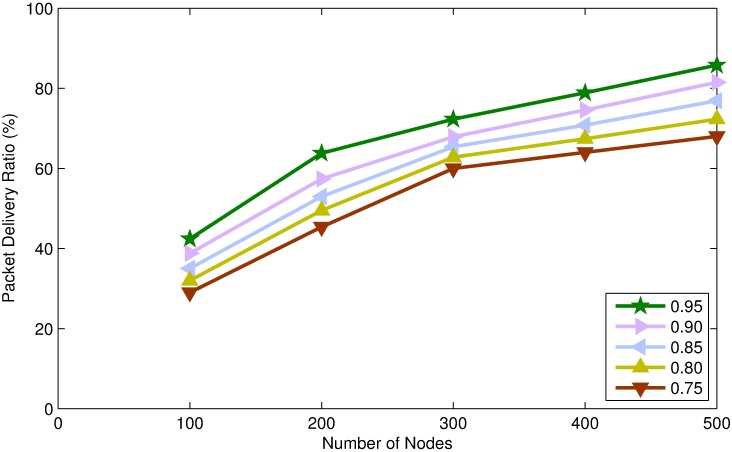
Packet delivery ratio vs. node density.

**Fig 7:**
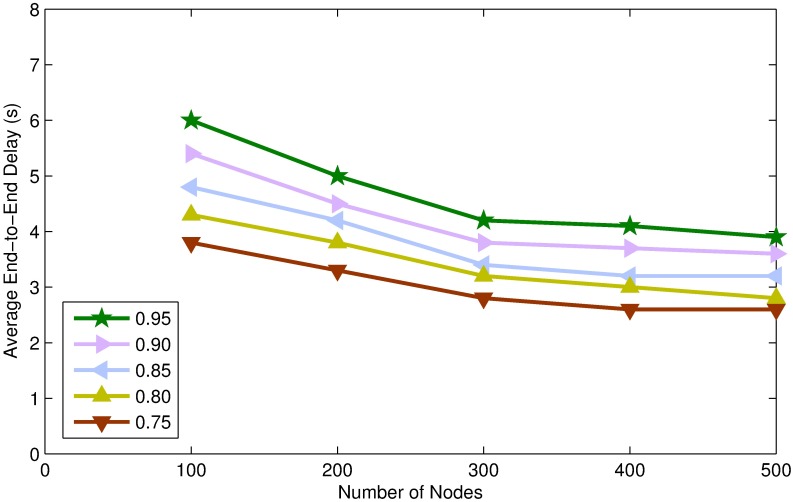
Average end-to-end delay vs. node density.

By assigning a higher value to confidence threshold, very few number of nodes will be able to become STN resulting in DTNs majority network, which will lead into increasing number of control packet transmission and causes more energy to spend on per node per packet. Moreover, increasing the number of DTNs will increase end-to-end delay because the source node has to wait for 2-hop *Ack* of every packet transmitted towards the destination. However, increasing the number of DTNs will guaranty packet transmission and will increase the packet delivery ratio, as can be seen in Figs 5–7.

On the other hand, smaller value of confidence threshold will make many nodes eligible to be STNs. By increasing the number of STNs will lead into lowering the energy consumption on per packet because STNs do not demand for *Ack* and thus the number of control messages transmission is reduced. Moreover, increasing the number of STNs will reduce end to end delay, because data packets are forwarded as soon as the candidate forwarder receives the packet. However, increasing the number of STNs will decrease the packet delivery ratio, because the forwarding nodes do not demand for *Ack* and the packet may be lost during transmission. Furthermore, there is no retransmission mechanism defined for STNs and which may lead into lowering the packet delivery ratio, as shown in Figs 5–7.

Average hop count and propagation deviation factor does not seem to be directly affected by changing confidence threshold value. However, reducing the confidence threshold value increases node’s localization, which may result in having more paths from source to destination and thus an optimal path with the least number of hops and propagation deviation factor can be selected.

## Performance evaluation

In this section, we present the performance evaluation of TORA against well know existing UWSN routing protocols VBF [15] HH-VBF [16], VAPR [18], and H2DAB [19]. These protocols are evaluated based on several performance metrics such as energy consumption, packet delivery ratio, average end-to-end delay, average hop-count and propagation deviation factor.

### Energy consumption

A node has to spend energy to senses the environment and send or receive packets. In this section, we calculate the energy cost of a packet on per hop to be relayed towards sink node.

**Fig 8:**
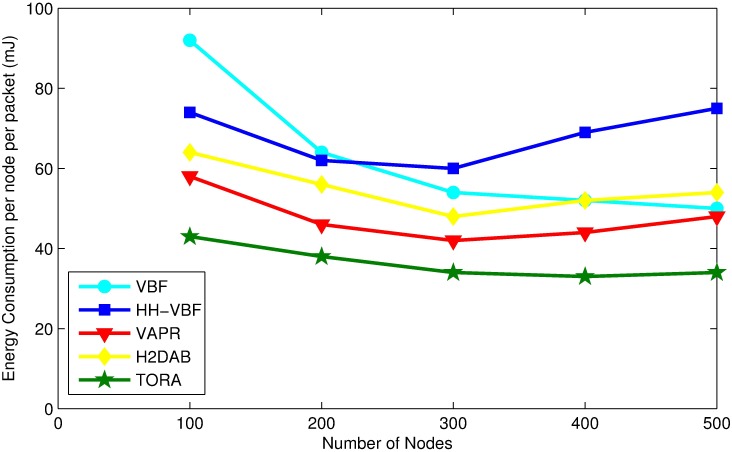
Energy consumption per node per message vs. node density.

Fig 8 shows the energy consumption for each protocol with the various number of nodes. TORA consumes the least energy for transmitting packets to the destination. The reason to this is that TORA confines candidate set transmissions by selecting the best available candidate forwarder, most probably a node locating closer to sink and maintaining higher residual energy will be the next candidate forwarder. Furthermore, TORA avoids packet collisions and prevents redundant transmissions which reduces energy consumption.

As the number of nodes increases, the overall energy consumption for most of the protocols also increases. It is due to the fact that more number of nodes are involved in data forwarding process. In contrast, in TORA the energy consumption is the lowest, because it allows limited number of nodes in candidate forwarder set. Only node with the highest priority, higher residual energy and closer to sink among neighbors has the shortest holding time and it forwards the packets as soon as it receives it. Upon overhearing of the packet transmission, all candidate forwarder holding a packet with the same sequence number drops the packet.

Energy consumption in VBF and HH-VBF based on the size of virtual pipe radius. If a greater value is set to radius of a virtual pipe then there will be many potential forwarder for a packet, which will increase packet delivery ratio but on the other hand it will also increase duplicate packet transmission, which is wastage of energy. On the contrary, selecting a smaller radius to virtual pipe may lead to network holes, which results in more frequent failure to packet transmission. In very sparse networks, the energy consumption of VBF and HHVBF is high due to the low packet delivery ratio of them.

In H2DAB significant amount of control messages are exchanged for assigning hop IDs and forwarding path selection in the form of inquiry request/reply packets. These packets consumes excessive energy and affect the network lifetime adversely. Moreover, similar to VBF, the nodes closer to sink are used more rapidly, which causes network holes. At each hop the inquiry request/reply messages are exchanged and by increasing the number of nodes the number of hops increases, which increase the energy consumption as well.

In comparison to VAPR, we found out that VAPR is not flexible enough to adjust the forwarding set regarding network density, which may affect its performance adversely and leads to energy depletion. Moreover, VAPR forces the nodes to calculate distances between neighboring nodes more frequently which leads to higher energy consumption.

### Packet delivery ratio (PDR)

**Fig 9:**
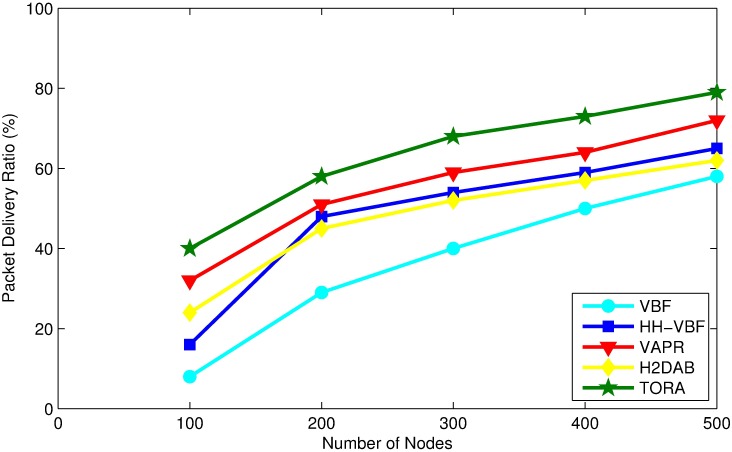
Packet delivery ratio vs. node density.

Packet delivery ratio (PDR) is the ratio of packets that are successfully received by a sink compared to the total number of packets that have been sent out by sender. The simulation results relating to PDR for the above mentioned protocols are presented in Fig 9. As can be seen from the figure, PDR increases with increase in the number of nodes, because it will not only reduce void areas but there will be more forwarding nodes to be added to the routing path, and consequently improves the PDR.

TORA produces higher PDR in comparison to other routing protocols, and this value keeps on growing as the network becomes dense. The main reason behind this fact is that TORA avoids transmission paths which lead into the void node. Moreover, the use of multiple nodes in candidate set and the utilization of packet delivery probability helps it to achieve higher packet delivery ratio. In most of the existing protocols a higher PDR is achieved with the expense of excessive energy consumption and increase in end-to-end delay. In contrast, TORA achieves higher PDR by assigning the best available node to forward the packet, and to insure packet delivery it uses 2-hop *Ack*. The use of 2-hop *Ack* helps TORA to avoid the void nodes, which improves PDR.

In VBF all the nodes residing in the routing vector are the potential candidate forwarder and which may cause high collision among data packets, which outcomes in low delivery ratio. Moreover, as soon as the void areas appear in their routing paths, the packet failure increases, which lowers its PDR. Furthermore, VBF does not consider packet delivery probability as a metric for candidate forwarder selection, which makes the neighboring nodes forward data packets to the void nodes more frequently. Simulation Results show that VBF is intended for a dense UWSN; On the contrary, HH-VBF has been designed for sparse network. Its performance improves as the number of nodes increases upto a certain extent and once the network becomes dense, its performance degrades gradually. However, it is notable that increasing the routing pipe width increases the delivery ratio.

H2DAB achieves low PDR due to the fact that all the data packets are routed on a single path from source to destination. If a packet drops at any intermediate node, there is no proper retransmission mechanism defined to forward the packet. Although, there is a retransmission mechanism in H2DAB, however, it selects the same forwarding node repeatedly due to selection criteria for next hop forwarder. Thus the packets may drop each time it is routed to a void node, which results in lowering packet delivery ratio. Apart from that, the implementation of retransmission mechanism is expensive and it results in higher energy consumption and end-to-end delay, which will adversely affect the performance of H2DAB.

VAPR is a multi-sink architecture based routing protocol, which produces higher PDR in comparison to VBF and HH-VBF, especially in the sparse network. However, it produces lower PDR than TORA, which could be obviously because of its inherited problem of two direction of transmission. VAPR can forward the packet in upward or downward direction only, which excludes horizontal transmission and that might affect the PDR adversely.

### Average end-to-end delay

**Fig 10:**
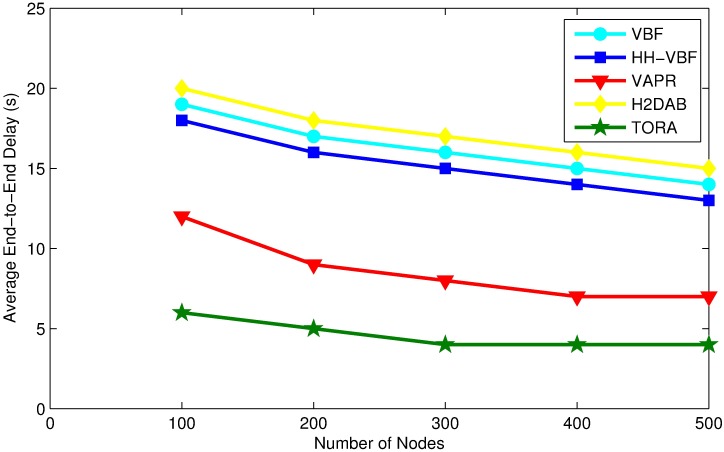
Average end-to-end delay vs. node density.

The average end-to-end delay is the time difference between packet generated at the source node and until it is successfully received at the sink node. end-to-end delay is the combination of transmission delay, propagation delay and holding time of a packet at a relay node. The average end-to-end delay for different protocols is shown in Fig 10.

As we increase the number of nodes, the average end-to-end delay tends to be decreasing. It is a matter of fact that in highly dense network there will be more potential forwarder for each packet, and the forwarding node can find an optimal path for packet transmission which will result in lowering the average of the end-to-end delay.

From Fig 10 it is apparent that TORA offers the best end-to-end delay performance compared to other protocols. The main reason behind this is the selection of best priority node on the fly and by assigning a very small holding time to a forwarding node. The highest priority node carries the smallest holding time and it forwards a packet as soon as it receives it. Moreover, TORA uses an optimal path from source to destination with least possible transmissions which reduces end-to-end delay.Furthermore, TORA reduces the number of collisions and retransmissions to the least amount possible, which improves packet delivery time.

However, in the case of VBF and HH-VBF a best-suited node may be located outside of the virtual pipe and would be ignored, that may increase the latency. The holding time for a relay node in TORA is obviously smaller than the desirability factor (pre-defined maximum delay) of VBF and HH-VBF. Furthermore, the selection of candidate set and prioritizing of candidate forwarder are entirely different in both schemes. The end-to-end delay for VBF and HH-VBF has also been adversely affected by the existence of hidden node within the virtual pipe, which increases the number of collisions and so as retransmissions. The average end to end delay for these protocol is higher than TORA due to different ways of selecting and prioritizing candidate forwarding set.

H2DAB based on inquiry request/reply for path selection and data forwarding. The sender broadcast inquiry request packet and wait for receiving the inquiry reply packet from all its neighbors. Thus the waiting time for receiving inquiry reply packet and long propagation delay of acoustic signal increases the end-to-end delay. In comparison to VBF, the end-to-end delay of H2DAB is higher because in VBF the next hop forwarder is selected based on distance between the forwarder and the routing vector. Although, VBF uses holding time at each hop, but that is small enough for shorter routing pipe. Meanwhile, the end-to-end delay in VBF increases with increasing the size of routing vector after a certain limit. During our simulation we set the routing pipe to 100m, where it performs the best with respect to end-to-end delay.

VAPR faces the problem of hidden nodes in its forwarding set and it’s not versatile enough to remove all of it, due to its inherited limitation of basing on estimated distance of 2-hop connectivity, which is not precise enough in comparison to TORA. On the other hand, TORA outperforms VAPR for the per-packet delay, because VAPR can forward a packet either in upward or downward direction. Even though it uses the reachability information but still it ends up with a higher end-to-end delay. In TORA, the number of collisions and retransmission has been minimized to the least possible amount, which improves the packet delivery time and reduces end-to-end delay.

### Average hop-count

Average hop-count is average number of hops that a packet travel to reach sink node. Involving the least possible number of nodes in a routing path is a desirable factor for all routing protocols. An efficient routing algorithm would strive to find an optimal route with fewer transmission from source to destination.

**Fig 11:**
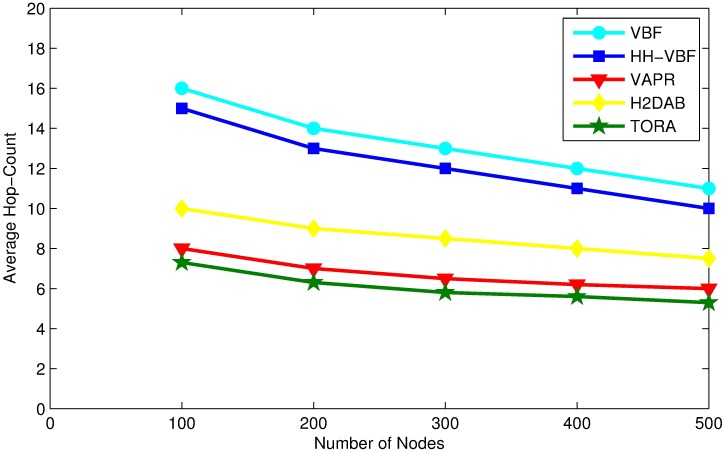
Average hop-count vs. node density.

The average number of hops to relay a packet to sink varies with network density. In the sparse network, there may be no forwarding node available on the shortest line between source and destination and the packet has to be routed on the longer path, which involves more relay nodes to transmit the packet to sink. However, In the case of the dense network the chances for finding the routing path with fewer node increases, which leads to lowering the average hop count, as can be seen in Fig 11.

TORA involves least intermediate node due to its flexibility in selecting candidate forwarder and utilizing the global view of network topology by 2-hop *Ack*. Evaluation results conclude that the density of the nodes minimally influence the average hop count for TORA.

VBF and HH-VBF are confined to the radius of the virtual pipe and they are not flexible enough to find a route from source to destination with the minimal number of intermediate nodes. Furthermore, in these protocols data forwarding priority is given to the nodes which are closer to the virtual pipe and not necessarily the nodes are on lower hop count distance to the sink. Although H2DAB, selects a path with less number of hop than VBF and HH-VBF but still it’s more than that of TORA. Moreover, the path discovered by VAPR has more number of intermediate nodes than TORA, because VAPR basis on the directional trail not by hop count.

### Propagation deviation factor (PDF)

**Fig 12:**
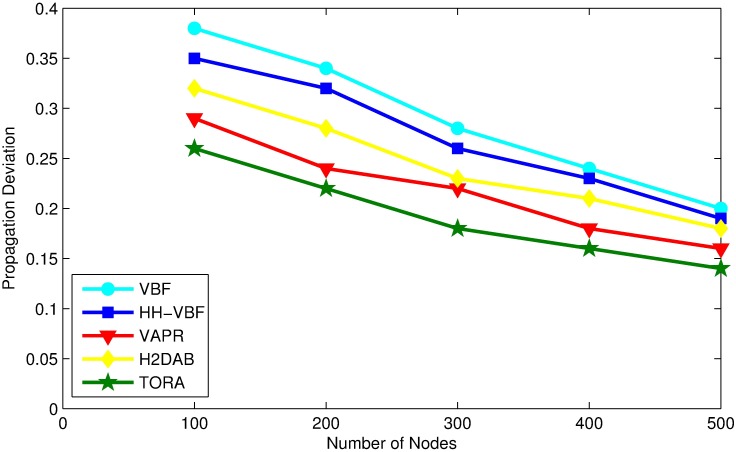
PDF vs. node density.

Propagation Deviation Factor (PDF) shows a normalized value of distance for each packet that travel from source to sink. It can be calculated as,

$$\begin{array}{c} PDF=\frac{D_{T}-D_{S}}{D_{S}} \end{array}$$ (19)

where *D*_*T*_ is the total distance traveled by a packet to reach destination and *D*_*S*_ is the straight line distance between source and destination. An ideal routing protocol will select the best available routing path along the straight line with lowest propagation deviation factor. The PDF will increase if the packet is routed on a longer path from source to destination. PDF for each of the protocol is presented in Fig 12. As can be seen, the PDF decreases with increasing number of nodes, because there will be shorter links to build an optimal routing path.

TORA got the lowest value of PDF in comparison to other routing protocols, which is due to the utilization of node actual coordinates and distance to sink in candidate forwarder selection, that makes it able to route packet with an optimal path and reduce the traversed distance.

On the other hand, VBF gets a good PDF in the dense network; however both VBF and HH-VBF obtains a higher PDF in the case of the sparse network because the routing path selected is far away from an optimal one. While H2DAB basis on hop number for routing path selection, which does not guarantee the exact location of nodes within network. To find an optimal path with least PDF it is important to know the nodes exact locations within network. Although, H2DAB, manages to find shorter routing paths with less PDF than VBF and HH-VBF but still it’s higher in comparison to TORA, which is due to the difference in selection criteria of forwarding nodes in both protocols. On the other hand VAPR avoids void regions in the routing path but it is not always interested in selecting the forwarding nodes, which are closer to the sink but routing paths are created on the basis of directional trails.

## Conclusion

This paper proposed a hierarchical localization scheme, and based on that we designed a novel anycast, receiver based opportunistic and geographical routing protocol for UWSN. Nodes are localized with the help of trilateration and TOA, and node location information along with its residual energy is used to find the best available forwarding node in a closer proximity to the destination. Thus multiple short and active links are combined to transmit data to the sink.

We studied, the effects of the channel characteristics on end-to-end delay, energy efficiency, and packet delivery ratio with various node density. Extensive simulations were performed to evaluate the performance of the proposed scheme in comparison to some existing related routing protocols. Simulation shows that TORA extends network lifetime by improving energy efficiency, increases packet delivery ratio while reduces end-to-end delay and propagation deviation factor.

In future, we plan to further extend and investigate our proposed scheme using the operations research techniques to find the optimal routes and forward data through these routes to the sink node. Moreover we plan to analyze and investigate the behavior of our proposed scheme using the Meandering Current Mobility model.

## References

[1] Akyildiz IF, Dario P, and Tommaso M. Underwater acoustic sensor networks: research challenges. Ad hoc networks 3.3 (2005): 257-279.

[2] Ayaz M, Baig I, Abdullah A and Faye I. A survey on routing techniques in underwater wireless sensor networks. Journal of Network and Computer Applications 34 6 (2011): 1908–1927. doi: 10.1016/j.jnca.2011.06.009

[3] Liu L, Ruchuan W, and Fu X. Topology control algorithm for underwater wireless sensor networks using GPS-free mobile sensor nodes. Journal of Network and Computer Applications 35 6 (2012): 1953–1963. doi: 10.1016/j.jnca.2012.07.017

[4] Das AP, and Thampi SM. Secure communication in mobile underwater wireless sensor networks. Advances in Computing, Communications and Informatics (ICACCI), 2015 International Conference on. IEEE, 2015.

[5] Zeng D, Wu X, Wang Y, Chen H, Liang K, and Shu L. A survey on sensor deployment in underwater sensor networks. China Conference wireless sensor networks. Springer Berlin Heidelberg, 2013.

[6] Jiang J, Han G, Guo H, Shu L, and Rodrigues JJPC. Geographic multipath routing based on geospatial division in duty-cycled underwater wireless sensor networks. Journal of Network and Computer Applications 59 (2016): 4–13.

[7] Akyildiz IF, Pompili D, and Melodia T. Challenges for efficient communication in underwater acoustic sensor networks. ACM Sigbed Review 1 2 (2004): 3–8. doi: 10.1145/1121776.1121779

[8] Wang K, Gao H, Xu X, Jiang J, and Yue D, An energy-efficient reliable data transmission scheme for complex environmental monitoring in underwater acoustic sensor networks IEEE Sensors Journal, vol. 16,no. 11, pp. 4051–4062, 2016 doi: 10.1109/JSEN.2015.2428712

[9] Biswas S, and Morris R. ExOR: opportunistic multi-hop routing for wireless networks. ACM SIGCOMM Computer Communication Review 35 4 (2005): 133–144. doi: 10.1145/1090191.1080108

[10] Boukerche A, and Darehshoorzadeh A. Opportunistic routing in wireless networks: Models, algorithms, and classifications. ACM Computing Surveys (CSUR) 47 2 (2015): 22.

[11] Boukerche A, Turgut B, Aydin N, Ahmand ZM, Boloni L, and Turgut D. Routing protocols in ad hoc networks: A survey. Computer networks 55 13 (2011): 3032–3080. doi: 10.1016/j.comnet.2011.05.010

[12] Darehshoorzadeh A, and Boukerche A. An efficient heuristic candidate selection algorithm for opportunistic routing in wireless multihop networks. Computers and Communication (ISCC), 2014 IEEE Symposium on. IEEE, 2014.

[13] Nowsheen N, Gour K, and Kamruzzaman J. An adaptive approach to opportunistic data forwarding in underwater acoustic sensor networks. Network Computing and Applications (NCA), 2014 IEEE 13th International Symposium on. IEEE, 2014.

[14] Han G, Jiang J, Bao N, Wan L, and Guizani M. Routing protocols for underwater wireless sensor networks. IEEE Communications Magazine 53 11 (2015): 72–78. doi: 10.1109/MCOM.2015.7321974

[15] Xie P, Cui JH, and Lao L. VBF: vector-based forwarding protocol for underwater sensor networks. International Conference on Research in Networking. Springer Berlin Heidelberg, 2006.

[16] Nicolaou N, See A, and Xie P. Improving the robustness of location-based routing for underwater sensor networks. OCEANS 2007-Europe. IEEE, 2007.

[17] Yan H, Jerry SZ, and Cui JH. DBR: depth-based routing for underwater sensor networks. International conference on research in networking. Springer Berlin Heidelberg, 2008.

[18] Noh Y, Lee U, and Wang P. VAPR: void-aware pressure routing for underwater sensor networks. IEEE Transactions on Mobile Computing 12 5 (2013): 895–908. doi: 10.1109/TMC.2012.53

[19] Ayaz M, Abdullah A, Faye I, and Batira Y. An efficient dynamic addressing based routing protocol for underwater wireless sensor networks. Computer Communications 35 4 (2012): 475–486. doi: 10.1016/j.comcom.2011.11.014

[20] Diao B, Xu Y, An Z, Wang F, and Li C. Improving both energy and time efficiency of depth-based routing for underwater sensor networks. International Journal of Distributed Sensor Networks. 11 10 (2015):781932.

[21] Ibrahim D, Tarek E, Mahmoud F, and Elsayed S. Enhancing the vector-based forwarding routing protocol for underwater wireless sensor networks: a clustering approach. The Tenth International Conference on Wireless and Mobile Communications (ICWMC). 2014.

[22] Leroy CC, and Parthiot F. Depth-pressure relationships in the oceans and seas. The Journal of the Acoustical Society of America. 103 3 (1998): 1346–1352. doi: 10.1121/1.421275

[23] Darehshoorzadeh A, and Boukerche A. Underwater sensor networks: A new challenge for opportunistic routing protocols. IEEE Communications Magazine 53 11 (2015): 98–107. doi: 10.1109/MCOM.2015.7321977

[24] http://obinet.engr.uconn.edu/wiki/index.php/Aqua-Sim.

